# A high-density consensus map of A and B wheat genomes

**DOI:** 10.1007/s00122-012-1939-y

**Published:** 2012-08-08

**Authors:** Daniela Marone, Giovanni Laidò, Agata Gadaleta, Pasqualina Colasuonno, Donatella B. M. Ficco, Angelica Giancaspro, Stefania Giove, Giosué Panio, Maria A. Russo, Pasquale De Vita, Luigi Cattivelli, Roberto Papa, Antonio Blanco, Anna M. Mastrangelo

**Affiliations:** 1 CRA-Cereal Research Centre, SS16 km 675, 71122 Foggia, Italy; 2 Department of Agro-Forestry and Environmental Biology and Chemistry, University of Bari, Via Amendola, 165/A, 70126 Bari, Italy; 3 CRA-Genomics Research Centre, Via S. Protaso 302, 29017 Fiorenzuola d’Arda, PC Italy

## Abstract

A durum wheat consensus linkage map was developed by combining segregation data from six mapping populations. All of the crosses were derived from durum wheat cultivars, except for one accession of *T.* ssp. *dicoccoides*. The consensus map was composed of 1,898 loci arranged into 27 linkage groups covering all 14 chromosomes. The length of the integrated map and the average marker distance were 3,058.6 and 1.6 cM, respectively. The order of the loci was generally in agreement with respect to the individual maps and with previously published maps. When the consensus map was aligned to the deletion bin map, 493 markers were assigned to specific bins. Segregation distortion was found across many durum wheat chromosomes, with a higher frequency for the B genome. This high-density consensus map allowed the scanning of the genome for chromosomal rearrangements occurring during the wheat evolution. Translocations and inversions that were already known in literature were confirmed, and new putative rearrangements are proposed. The consensus map herein described provides a more complete coverage of the durum wheat genome compared with previously developed maps. It also represents a step forward in durum wheat genomics and an essential tool for further research and studies on evolution of the wheat genome.

**Electronic supplementary material:**

The online version of this article (doi:10.1007/s00122-012-1939-y) contains supplementary material, which is available to authorized users.

## Introduction

Durum wheat [*Triticum turgidum* (L.) subsp. *turgidum* (L.) convar. *durum* (Desf.)] is characterized by a large allotetraploid genome (AABB genome, 2*n* = 4*x* = 28, seven homoeologous groups—13,000 Mbp). Although durum wheat accounts for about 10 % of the total wheat production (World Grain Statistic, http://www.igc.org.uk), it is particularly important for its end products, which are mainly pasta, couscous and bulgur. Intense breeding activities have been carried out over the past century to improve the durum wheat varieties in terms of grain yield and quality, disease resistance, and drought tolerance (De Vita et al. 2007). Plant breeding is a long-term process and molecular tools can be used to overcome difficulties and to open the way for more rapid and efficient breeding strategies (Gupta et al. 2008; Tester and Langridge 2010). The phenotypic variation of many complex traits of agricultural or evolutionary importance is influenced by quantitative trait loci (QTL), their interactions, the environment, and the interactions between the QTL and the environment. Linkage mapping has been largely adopted in wheat to identify genomic regions that are involved in the control of complex traits (Breseghello and Sorrells 2006; Kuchel et al. 2007; Gupta et al. 2008), and many genetic maps of durum wheat have been published (Blanco et al. 1998; Nachit et al. 2001; Elouafi and Nachit 2004; Zhang et al. 2008; Peleg et al. 2008; Mantovani et al. 2008; Gadaleta et al. 2009). The early maps were based on restriction fragment length polymorphism (RFLP) markers (Blanco et al. 1998), while later the polymerase chain reaction (PCR)-based markers became dominant for genetic map construction, e.g. amplified fragment length polymorphisms (AFLPs) (Nachit et al. 2001) and simple sequence repeats (SSRs) (Peleg et al. 2008; Gadaleta et al. 2009). More recently, single-nucleotide polymorphisms (SNPs) have been included in durum wheat genetic maps (Zhang et al. 2008; Trebbi et al. 2011). The availability of SSR markers for durum wheat (Eujayl et al. 2002) and the development of high-throughput systems such as diversity array technology (DArT) (Jaccoud et al. 2001) have overcome the difficulties of genotyping large panels of genotypes with many loci. DArT technology in particular provides a highly multiplexed platform, which allows for rapid and cost-effective genome-wide genotyping (Wenzl et al. 2004; Akbari et al. 2006).

The construction of integrated maps provides the opportunity to increase the marker coverage with respect to individual maps. Consensus maps have been developed in many plant species: bread wheat (Somers et al. 2004), barley (Wenzl et al. 2006), rye (Gustafson et al. 2009), soybean (Hwang et al. 2009), red clover (Isobe et al. 2009), and ryegrass (Studer et al. 2010). In *Vitis Vinifera* L. (Vezzulli et al. 2008) and durum wheat (Trebbi et al. 2011), integrated maps have allowed new SNP markers to be mapped (501 and 157, respectively). The importance of the construction of consensus maps relies on the development of genetic tools that provide an essential basis for further genomic research.

Structural rearrangements revealed by colinearity failures among homoeologs can be genetically characterized with linkage maps. In wheat specific chromosome rearrangements have been documented in the A, B, and D genomes, e.g. the cyclic translocation involving chromosomes 4A, 5A, and 7B (Blanco et al. 1998). An integrated genetic map with high marker density can be useful to scan the whole genome for different kinds of chromosomal rearrangements as translocations, inversions, and duplications.

The main aim of the present study was to develop a high-density durum wheat consensus map derived from the integration of six individual maps, as a reference resource for durum wheat scientists in molecular breeding programs, as well as for comparative genomics within grass species. Along with the consensus map, the assignment to deletion bin map of many markers is herein reported. Regions with segregation distortion were identified by combining data from the single populations. Finally, an extensive analysis of multi-locus markers has allowed the identification of numerous chromosomal rearrangements.

## Materials and methods

### Segregating populations and genetic maps

A total of six mapping populations, developed to serve specific needs for qualitative and quantitative trait analysis, were used to integrate nearly 2,000 unique loci into a single consensus map: ‘Creso’ × ‘Pedroso’ [CP, 123 recombinant inbred lines (RILs) F_8_–F_9_], ‘Ofanto’ × ‘Cappelli’ (OC, 161 RILs F_8_–F_9_), ‘Cirillo’ × ‘Neodur’ (CN, 178 RILs F_8_–F_9_), ‘Ciccio’ × ‘Svevo’ (CS, 120 RILs F_7_–F_8_), ‘Latino’ × ‘Primadur’ (LP, 121 F_2_–F_3_ families), and ‘Messapia’ × ‘MG4343’ (MM, 65 RILs F_7_–F_8_). All of these genotypes are durum wheat varieties, except MG4343, which is an accession of *Triticum* *turgidum* (L.) sub-species *dicoccoides*.

The genetic map obtained from the MM population represented the first map to be constructed in tetraploid wheat (Blanco et al. 1998). This was achieved mainly with RFLP markers, then the map was enriched with SSRs (Blanco et al. 2004), and this version was used for the development of the consensus map. The CP population was used to study the genetic basis of durable leaf rust resistance of the cultivar Creso (Marone et al. 2009) and then implemented with additional 75 microsatellite markers (Marone et al., personal communication). The map derived from the OC population was developed to find chromosomal regions involved in the response to drought stress, as these two cultivars have different water-use efficiencies (Rizza et al. 2012). Furthermore, both CP and OC were used to map genes coding for different lipoxygenase isoforms on chromosome 4B (Verlotta et al. 2010). The CN map allowed the identification of a major and some minor QTL that explain the resistance against soil-borne cereal mosaic virus in the Neodur variety (Russo et al. 2011). As the CN map was implemented after the beginning of the work on consensus map, the dataset used in the present study is smaller than the one used by Russo et al. (2011) (290 vs. 426 markers). The CS map was developed for genetic and physical mapping of new expressed sequence tag (EST)-SSRs (Gadaleta et al. 2009), and it was then enriched with DArT markers to identify loci that are involved in seed protein content (Blanco et al. 2012). Finally, the LP population was used to determine the genetic basis of yellow pigment content and carotenoid accumulation (Blanco et al. 2011).

The main features of the segregating populations and the corresponding genetic maps are reported in Table 1, whereas Online resource 1 reports the different sources of markers used for the construction of the maps.

**Table 1:** Summary of the six mapping populations used to construct the consensus map of durum wheat

Parents	Population size	Markers				Total markers	Map length (cM)	Marker density (cM/marker)	Reference
		SSR	EST-derived (SSR,STS)	DArT	Other markers^a^				
‘Creso’ × ‘Pedroso’	123	191	44	340	–	575	2,221.3	3.8	Marone et al. (2009)
‘Ofanto’ × ‘Cappelli’	161	154	23	437	4	618	1,649.4	2.6	Verlotta et al. (2010)
‘Cirillo’ × ‘Neodur’	178	71	7	212	–	290	1,568.5	5.4	Russo et al. (2011)
‘Ciccio’ × ‘Svevo’	120	132	110	584	4	830	1,765.8	2.1	Gadaleta et al. (2009)
‘Latino’ × ‘Primadur’	121	96	22	322	–	440	1,066.2	2.4	Blanco et al. (2011, 2012)
‘Messapia’ × ‘MG4343’	65	84	–	–	356	440	2,913.2	6.6	Blanco et al. (2004)

^a^RFLP, TRAP, biochemical and morphological markers

### Construction of the consensus map

JoinMap 4.0 software (Van Ooijen and Voorips 2004) was used to reproduce the six durum wheat genetic maps and to generate the consensus map. The significance of deviations of the observed allelic frequencies from the expected ratios (1:1 or 1:2:1) (*P* < 0.01) was tested using Chi-squared analysis. The segregation data of each mapping population were first analyzed chromosome by chromosome, using a minimum logarithm of odds (LOD) score of 4 for grouping. The Kosambi mapping function (Kosambi 1943) and the “fixed order” of marker loci were used to reproduce linkage groups that correspond to the single maps previously developed. Subsequently, the linkage groups for each chromosome derived from the six mapping populations were joined using the “combine groups for map integration” function within the JoinMap software. When necessary, markers were removed from the analysis (i.e. markers with too much missing data), and the order was recalculated, until a stable and consistent order was obtained with respect to the single genetic maps. For some markers on chromosome 7B the order of consensus loci reproduced by the software was not consistent with that observed in individual maps. In this case, a fixed order was imposed based on data consistency in more than one individual map. The centromeres were positioned onto the consensus map at the midpoints between the most proximal markers on the short and long arms, according to common markers between this map and those of Röder et al. (1998), Somers et al. (2004) and Gadaleta et al. (2009).

**Fig. 1:**
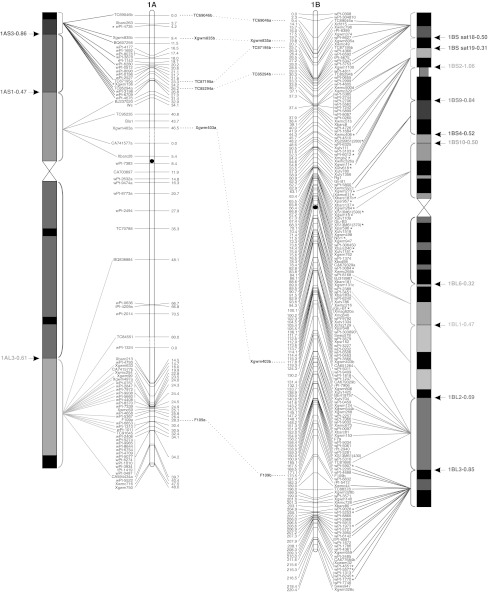

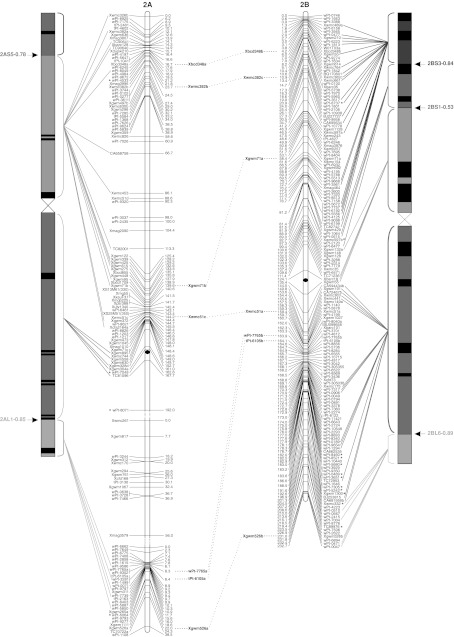

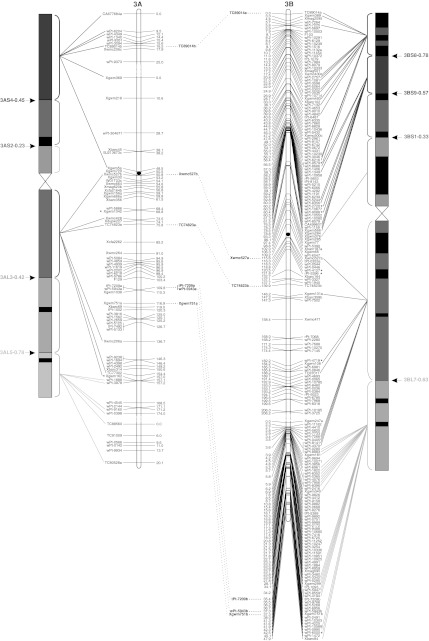

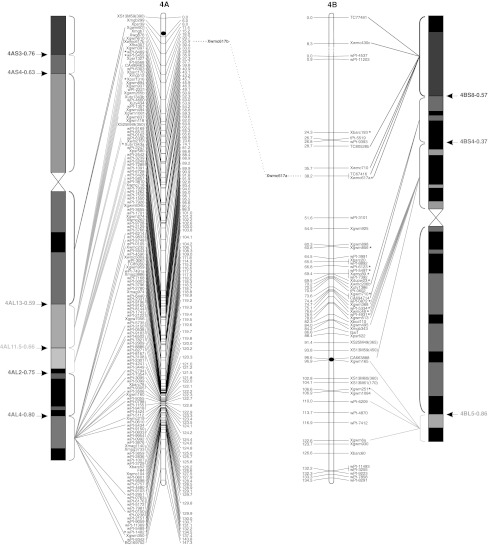

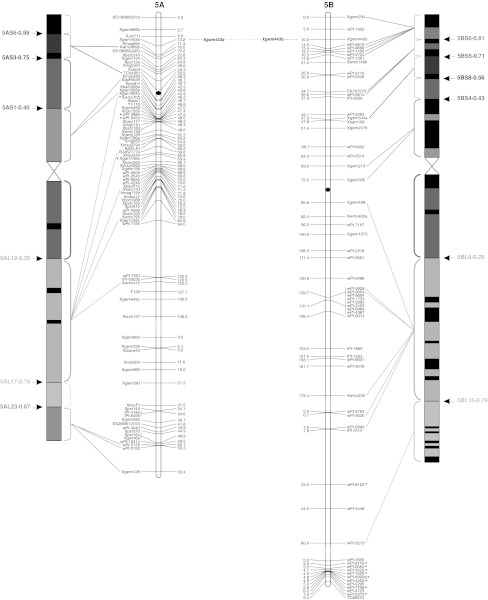

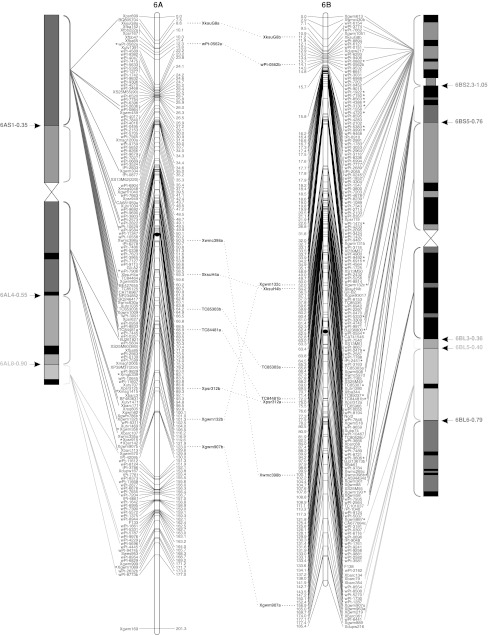

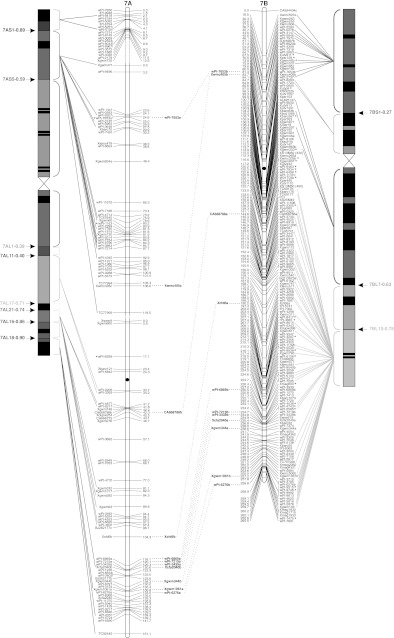
The durum wheat consensus linkage map. The deletion bin map as reported by Gadaleta et al. (2009) was aligned with the consensus map and *the colored lines* show the genetic/physical relationships for each marker. *Straight lines* connect markers to specific bins, characterized by different colors. *Dotted lines* connect homoeologous loci that are reported between the two chromosomes of each pair. Markers with segregation distortion (*P* < 0.01) are marked with an *asterisk*. Markers identifying two or more mapped loci have the suffix “.a”, “.b”, and so on. The centromeres are indicated by *black circles*. *Dashed* *lines* on chromosomes indicate break points (color figure online)

To validate the marker order of the consensus map, genomic SSR, EST-SSR, and DArT markers were assigned to specific deletion bins when possible, using the resources available. The physical positions of genomic SSR and EST-SSR markers were obtained by Francki et al. (2008) and Gadaleta et al. (2009), whereas for DArT markers this was determined by Francki et al. (2008) and by the deletion bin maps available at http://www.cereals.uk.net. Figure 1 reports the physical map described by Gadaleta et al. (2009) based on a set of 58 common wheat deletion lines dividing the A and B genome chromosomes into 94 bins (Endo and Gill 1996), in which physical mapping data derived from different sources were integrated.

Once developed, the consensus map was compared with the six individual maps and with the bread and durum wheat maps available in the literature (Röder et al. 1998; Somers et al. 2004; Francki et al. 2008; Gadaleta et al. 2009; Trebbi et al. 2011), in terms of marker order and genetic marker distance.

### Analysis of colinearity between homoeologous chromosomes and structural rearrangements

Multi-locus markers were considered, and loci revealed by the same marker were inspected to identify homoeologous and paralogous loci. Chromosomal rearrangements were analyzed by studying orthologous loci mapped on non-homoeologous groups and by evaluating the information available in the literature on the position of all markers included in the putative translocated regions.

## Results

### Overview of the individual linkage maps

The six individual genetic maps differ according to the types and the numbers of markers, the lengths, and the marker densities of the linkage groups; their main features are summarized in Table 1.

The individual maps carried between 290 (CN) and 830 (CS) loci that are assembled into a number of linkage groups, ranging from 19 (LP) to 37 (CS). The marker density was from 2.1 cM/marker (CS) to 6.6 (MM) cM/marker. The highest proportion of markers (24 %) was positioned on chromosome 3B in CS, whereas the lowest (1.3 %) was mapped on chromosome 5A in CN. Generally, the homoeologous groups 3, 6, and 7 contained higher percentages of loci in most of the analyzed populations. On the contrary, the homoeologous groups 1 and 5 were characterized by a lower marker density. The percentages of mapped markers were higher on the B than the A genome (A genome: 38–45 % vs. B genome: 55–62 %), except for MM, where 55 % of the markers were mapped on the A genome. The lengths of the resulting maps varied between 1,066 cM (LP) and 2,913 cM (MM). Gaps with genetic distances greater than 20 cM were found in all of these linkage maps.

### Construction of the consensus map

The durum wheat consensus map is composed of 1,898 loci (1,185 DArT, 388 genomic SSR, 166 EST-derived loci, and 159 other markers, e.g. RFLP, TRAP, biochemical and morphological loci) arranged into 27 linkage groups covering all 14 chromosomes (Table 2; see Online resource 2). A total of 650 mapped markers (216 PCR-based and 434 DArT) were common among at least two of the mapping populations (Table 3), while a total of 1,397 markers were unique to a specific mapping population. The total length of the integrated map was 3,058.6 cM. The mean length of the 27 linkage groups was 218.4 cM, although the chromosome size ranged from 142.7 cM (4B) to 286.5 cM (2A) (Table 2). The number of markers for each chromosome ranged from 58 (4B) to 261 (3B), with an average of 136.8. The average marker distance per chromosome was between 0.9 cM (4A and 3B) and 3.9 cM (5B), with an average density of one marker per 1.6 cM for the whole genome.

**Table 2:** Density and distribution of markers in the consensus map of durum wheat

Chromosome	Markers				Total markers	Map length (cM)	Marker density (cM/marker)
	DArT	SSR	EST-derived (SSR, STS)	Other^a^			
1A	50	13	15	2	80	174.5	2.2
2A	61	44	12	10	127	286.5	2.3
3A	37	23	11	1	72	219.1	3.0
4A	105	27	8	14	154	147.2	0.9
5A	16	34	10	23	83	200.9	2.4
6A	96	21	19	27	163	201.3	1.2
7A	80	22	9	–	111	283.2	2.5
Genome A	445	184	84	77	790	1,512.7	1.9
1B	84	45	17	27	173	220.4	1.2
2B	112	35	15	2	164	232.7	1.4
3B	229	24	8	–	261	254.1	0.9
4B	22	21	6	9	58	142.7	2.4
5B	48	12	2	–	62	245.3	3.9
6B	119	25	16	16	176	185.4	1.0
7B	126	42	18	28	214	265.5	1.2
Genome B	740	204	82	82	1,108	1,545.9	1.4
Total	1,185	388	166	159	1,898	3,058.6	1.6

^a^RFLP, TRAP, biochemical and morphological markers

**Table 3:** Common markers across mapping populations

Common markers between	Markers				Total common markers between populations
	DArT		PCR-based		
	Genome A	Genome B	Genome A	Genome B	
Two populations	102	171	77	70	420
Three populations	64	70	16	27	177
Four populations	12	15	12	12	51
Five populations	–	–	2	–	2
Total	178	256	107	109	650

The approximate location of the centromere was identified (Fig. 1) based on the integration of bread and durum wheat linkage (Röder et al. 1998; Somers et al. 2004; Gadaleta et al. 2009) and physical maps (Francki et al. 2008; http://www.cerealsdb.uk.net). Most markers were relatively evenly distributed along the chromosomes in terms of their genetic distances, although some regions were characterized by higher or lower marker densities (Fig. 1). The largest gap (that is a big genetic distance between two adjacent markers of the same linkage group) was 26.4 cM, between *wPt*-*7185* (94.0 cM) and *wPt*-*7201* (120.4 cM) on chromosome 5A of the consensus map. Additional gaps (more than 20 cM) were present on chromosomes 2A, 6A, 7A, 4B, and 5B. Some of the gaps of the individual maps were also present in the consensus map, whereas others were filled by integrating the information derived from different populations. For example, the 25 cM gap in CN on chromosome 4A (between *wPt*-*6330* and *wPt*-*1262*) is well covered by markers in the consensus map.

The order of loci of the consensus map was in good agreement with the corresponding orders of the individual linkage maps. Some exceptions concerned changes that occurred within a small interval (<10 cM). Considering the relative marker distances, the largest discrepancy was observed between markers *Xwmc716* and *Xbarc213* on chromosome 1A, where the genetic distance was 30 cM in the consensus, while it was only 7 cM in CP.

The position of the loci mapped in this study was compared with recently published maps of bread and durum wheat. The ITMI map (Song et al. 2005) and the consensus map developed by Somers et al. (2004), which represent two well-saturated bread wheat maps, were considered for SSRs, while the bread and durum wheat maps described by Crossa et al. (2007), Peleg et al. (2008), and Trebbi et al. (2011) were taken into account for comparisons of both the SSR and DArT marker positions. The genetic positions of most SSR and DArT loci in the durum wheat consensus map showed consistency with their positions in the reference maps with few exceptions. In some cases, differences in the relative distances between two markers were found, although these were not considered as real discrepancies as they involved markers that identified multiple loci, with paralogous loci mapped on the same chromosome. As an example, the markers *Xgwm443* and *Xgwm666* were positioned on chromosome 5A at a distance of about 120 cM by Somers et al. (2004) and Song et al. (2005). Three loci were mapped in the durum wheat consensus map for *Xgwm443*: the *Xgwm443b* locus was located on chromosome 5B, while the *Xgwm443a* and *Xgwm443c* loci were located on chromosome 5A. *Xgwm443a* was 13.2 cM from *Xgwm666*, but *Xgwm443c* was positioned at 130.5 cM according to the bread wheat consensus map.

The marker order is in agreement with the maps published by Somers et al. (2004), Song et al. (2005) and Trebbi et al. (2011) for ten chromosomes (1B, 2A, 3A, 3B, 4A, 4B, 5A, 5B, 6A, and 7A), while some inconsistencies were seen for the remaining chromosomes.

A distance of about 40 cM was reported between the markers *Xgwm497* and *Xgwm99* by Somers et al. (2004) and Song et al. (2005) on the long arm of chromosome 1A, while the two markers were co-segregating on our consensus map. Nevertheless, the positions of these two markers in two individual maps (CS, 3 cM, and LP, 1 cM) supported the distances found in the consensus map, which was also confirmed by the durum wheat map reported by Elouafi and Nachit (2004), where the two markers were positioned 6 cM apart.

On the long arm of chromosome 1A, a group of DArT markers (which contained *wPt*-*6754* and *wPt*-*8644*) were positioned more than 40 cM distal from the marker *Xwmc716* by Trebbi et al. (2011), while in our consensus map, the marker *Xwmc716* was 13 cM distal from the same group of DArT markers. Even if this region was contributed only by the CP individual map, the distances in our consensus map for the markers *Xwmc716* and *wPt*-*8644* agree with findings reported by Peleg et al. (2008).

On chromosome 2B, an inconsistency was found for the region between markers *Xwmc149* and *wPt*-*6643*, which were localized in the telomeric region of the long arm by Trebbi et al. (2011), and in the pericentromeric region in our map. However, the detailed analysis of genetic marker distances within this interval in the individual maps validated the orientation reported here.

Another discrepancy in the order of the loci was observed between our durum wheat consensus map and previous reports for chromosome 6B for DArT markers. The marker *wPt*-*7935* was mapped near the centromere, at <2 cM from the marker *Xgwm193*, whereas Trebbi et al. (2011) mapped the two markers at a distance of 60 cM; however, the two markers were placed 2 cM apart by Peleg et al. (2008). Finally, the marker *wPt*-*0530* co-segregated with *Xgwm344* in the map of Trebbi et al. (2011) on the long arm of chromosome 7B, while a distance of 20 cM was found between the two markers in our consensus map, and a similar distance (13 cM) was reported by Crossa et al. (2007).

### Comparison of marker positions between the consensus and deletion bin maps

Four hundred and ninety-three markers (25.9 %; 256 DArTs, 153 gSSRs, and 83 EST-derived markers) were assigned to specific bins of the wheat deletion bin map (Fig. 1). Sixty-six of the 94 bins were covered by at least one marker, and the number of markers per bin ranged from 1 to 47, with a mean of 7.5. Except for the short arm of chromosome 4A, represented by only one molecular marker, the bin coverage with genetically mapped molecular markers was relatively good. For instance, all of the bins of chromosome 2B were covered by markers genetically mapped in the consensus map. These findings allowed anchoring between the consensus and physical maps of the durum wheat genome. A few discrepancies were noted. A group of nine DArTs (*wPt*-*4533*, *wPt*-*4197*, *wPt*-*5647*, *wPt*-*0102*, *wPt*-*3611*, *wPt*-*0277*, *wPt*-*7626*, *wPt*-*9624,* and *wPt*-*5839*) were genetically mapped on a region of chromosome 2AS between markers physically positioned in the pericentromeric region in the consensus map, while they were previously located to the short arm of the same chromosome (http://www.cerealsdb.uk.net). The marker *wPt*-*3566*, which was mapped to the centromeric region of chromosome 1B by Francki et al. (2008), was instead positioned on the long arm in the consensus map. On the same chromosome, based on the physical positions of the surrounding markers, the marker *Xbarc8* was assigned to the bin 1BS2-1.06, while it was physically mapped to the bin 1BS10-0.00-0.50 by Gadaleta et al. (2009). In all of these examples, the map positions in the consensus map for these markers were in agreement with those reported by Trebbi et al. (2011).

The physical positions on chromosome 3A of DArTs *wPt*-*5084*, *wPt*-*4859*, *wPt*-*1562,* and *wPt*-*2659*, which were previously mapped to the bin 3AL5-0.78-1.00 (http://www.cerealsdb.uk.net), corresponded to the centromeric region in the consensus map. In this case, the genetic positions of these markers were supported by very good agreement between the CP and OC individual maps.

On chromosome 5A of the consensus map, the markers *Xgwm186*, *Xbarc165,* and *Xbarc100* were on the long arm instead of the centromere, as described by Francki et al. (2008). Nevertheless, two loci were physically mapped by Sourdille et al. (2004) for the marker *Xgwm186*, in the centromeric region and the long arm, while the marker *Xbarc100* was assigned to 5AL in the same study.

The marker *Xbarc3*, which was previously physically positioned in the bin 6AS1-0.35-1.00 (Francki et al. 2008), was genetically mapped in the pericentromeric region on 6AS in the consensus map, as according to Somers et al. (2004) and Goyal et al. (2005). Similarly, the marker *CA668788b* that was previously positioned in the pericentromeric region of chromosome 7AS (Gadaleta et al. 2009) was mapped on chromosome 7AL in the consensus map. This was supported by the genetic position of the marker, which was highly consistent in two individual maps (OC and CS).

Finally, *Xwmc479* was previously mapped in the bin 7AS1-0.89 (Gadaleta et al. 2009), while in the consensus map its position is between *Xgwm471* and *Xgwm60*, and it is physically mapped in the 7AS5-0.59 bin (Francki et al. 2008; Xue et al. 2008). This genetic position is as according to Somers et al. (2004) and Xue et al. (2008).

Of note, some genetically close markers were instead located physically in distant bins. For example, *Xgwm5* and *Xwmc527b* were at a distance of only 2.3 cM on chromosome 3A, but they were physically mapped on the bins 3AS4-0.45-1.00 and 3AL3-0.00-0.42, respectively. Similar data were shown for this region by Somers et al. (2004). Analogous cases were observed on chromosome 4B for the markers *CA663888* and *Xgwm165*, at a distance of only 0.3 cM, but mapped physically in the bins 4BS4-0.00-0.37 and 4BL5-0.86-1.00, respectively, and on chromosome 6A, where the co-mapping markers *wPt*-*0357* and *BJ261821* were instead mapped physically on the short and the long arms, respectively.

### Segregation distortion

The percentage of skewed markers (*P* < 0.01) was different across the populations, varying from 0.6 to 11.8 % for CS and OC, respectively. Both co-dominant (SSR, STS, RFLP) and dominant (DArT) markers were subjected to deviation from the expected Mendelian 1:1 and 1:2:1 ratios. The distribution of the markers with segregation distortion was not uniform across chromosomes. Chromosomes 7B (OC) and 6B (LP) had 26 and 20 skewed markers, respectively, positioned in regions spanning less than 40 cM, and these were the chromosomes with the highest proportion of skewed markers. Chromosome 1A was the least affected by segregation distortion in all of the individual maps. Moreover, clusters of markers with skewed segregation were identified in all of the individual maps (data not reported).

A total of 149 markers (7.8 %) showed distorted segregation (*P* < 0.01) on the consensus map (Fig. 1). A similar ratio of skewed markers was found for DArT and SSR markers (7.3 and 7.9 %, respectively). Markers with segregation distortion were spread across all of the durum wheat chromosomes. Nevertheless, a statistically significant difference (*P* = 0.0032) was seen between the A and B genomes for the number of skewed markers: 120 markers on B and 29 on A (Fig. 1). The percentages with respect to the total number of markers positioned on each genome were 10.7 and 3.7 %, respectively. Considering all of the pairs of homoeologous chromosomes, the number of skewed markers was higher for those belonging to the B genome. The difference between homoeologs was low for groups 3 and 5, but generally high for the other pairs, e.g. on chromosome 1 there were two skewed markers (0.2 %) on 1A, compared with 30 skewed markers (17 %) on 1B.

Also in the consensus map skewed markers defined particular chromosome regions with distorted segregation, which therefore putatively contain loci involved in this phenomenon. In some cases, the skewed markers were spread over large chromosome regions, as seen for chromosomes 1A, 2A, 3A, 3B, and 7B. Single skewed regions were identified on chromosomes 4BS (pericentromeric region), 2BL and 5BL (telomeric region), while two regions were identified on chromosomes 1B (close to the centromere and close to the telomere of the long arm), and 6B (on the short arm and in the pericentromeric region). Finally, chromosome 7B showed a high number of skewed markers that were clustered in three regions all along the chromosome.

The clusters of skewed markers derived specifically from a single population, except for one region on chromosome 1B (from 44.3 to 100.1 cM), found to carry skewed markers in both MM and CP (see Online resource 3).

### Analysis of colinearity between homoeologous chromosomes and structural rearrangements

Multi-locus markers were mapped in the present study, which we define as markers based on the same primer pair or clone that identified more than one locus.

A total of 214 loci were produced by 94 multi-locus markers, and out of these, 82 (56 SSRs, 20 DArTs and 6 RFLPs) were mapped on homoeologous chromosomes, whereas 132 (105 SSRs, 18 DArTs, 6 RFLPs, and 3 TRAPs) were assigned to intra-chromosome or non-homoeologous inter-chromosome positions (see Online resource 4). The colinearity between chromosomes within homoeologous groups was well conserved, as shown in Fig. 1, except for some markers. Six markers (4 EST-SSRs and 2 SSRs) that mapped to homoeologous sites were identified for homoeologous group 1. The same order and genetic positions characterized all of the loci, with the exception of *Xgwm403*, which showed a locus on the short arm of chromosome 1A and another one on the long arm of chromosome 1B.

Fourteen homoeologous loci (10 SSRs and 4 DArTs) were identified for group 2. Their order was highly consistent, although three markers (*Xbcd348*, *Xwmc382,* and *Xgwm71*) were mapped on the pericentromeric region of the short arm of chromosome 2A and on the telomeric region of the short arm of chromosome 2B. Furthermore, two DArT markers (*wPt*-*7765* and *tPt*-*6105*) were located on the telomere of chromosome 2AL and on the centromeric area of chromosome 2BL.

A comparable number of homoeologous loci was found for group 3 (8 SSRs and 4 DArTs). There was consistent order and genetic positions of the markers along the chromosomes, except for the loci *tPt*-*7209*, *wPt*-*5943,* and *Xgwm751*, which mapped near to the centromere on chromosome 3AL and on the long arm of chromosome 3B. Only two homoeologous loci were mapped on both groups 4 and 5. While for group 5 the genetic position of the loci *Xgwm443* on chromosomes 5AS and 5BS showed perfect correspondence, *Xwmc617* was mapped in the pericentromeric region on chromosome 4AL and on the short arm of chromosome 4B. The same results were reported by Röder et al. (1998) for the marker *Xgwm165*, positioned on chromosomes 4AS and 4AL.

Nine markers (5 SSRs, 3 RFLPs, and 1 DArT) detected homoeologous loci on both chromosomes of group 6. The correspondence in terms of genetic position and marker order was good, except for *Xpsr312*, the loci of which were positioned around the centromere on chromosome 6AL and on the short arm of chromosome 6B, *Xgwm132*, which mapped on the long arm of chromosome 6A and on the short arm of chromosome 6B, and *Xwmc398*, for which two loci on chromosomes 6AS and 6BL were found. Finally, 22 homoeologous loci (12 SSRs and 10 DArTs) were identified on chromosomes 7A and 7B, showing colinearity between the chromosomes.

**Fig. 2:**
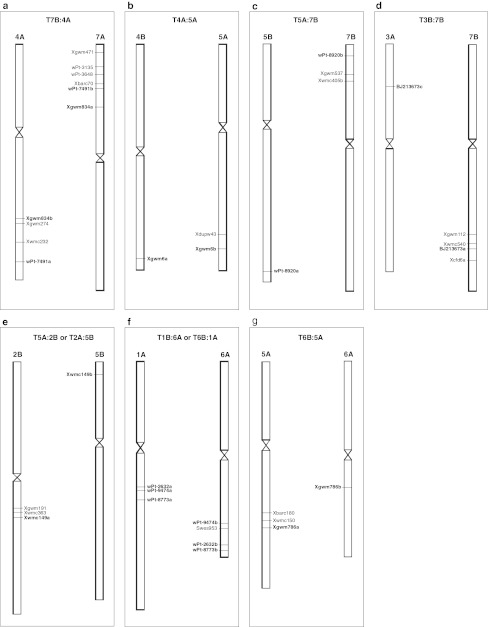
Genetic mapping of putative translocations in durum wheat. For each translocation the chromosome pair is reported with the molecular markers that identified non-homoeologous loci (*bold* *characters*). Markers positioned on one chromosome in the durum consensus map are in *normal* *characters*, for which additional loci on the other chromosome are reported in literature

Different chromosomal rearrangements have occurred during wheat evolution, such as duplications, inversions, and translocations. The construction of the consensus map reported in the present study with high number of markers and marker density allowed the wheat genome to be scanned for identification of such rearrangements. Groups of multi-locus markers that had loci mapped on non-homoeologous positions were considered to be involved in putative translocations. Then the markers included in these regions were evaluated in terms of their genetic positions reported in the literature (Fig. 2).

The markers *wPt*-*7491* and *Xgwm834* identified loci on chromosome 7AS and on the long arm of chromosome 4A, instead of chromosome 7B, as shown in Fig. 2a. Furthermore, *Xbarc70*, which mapped in the same region of chromosome 7AS, showed an additional locus on chromosome 4AL in OC (data not shown). Three additional markers (*wPt*-*3648*, *wPt*-*3135,* and *Xgwm471*), for which a single locus on chromosome 7AS was identified in the consensus map, were previously located on chromosome 4A (Song et al. 2005; Francki et al. 2008; Jing et al. 2009). Taken together, these data suggest that a translocation event took place between homoelogous groups 4 and 7. In addition, two markers (*Xgwm274* and *Xwmc232*) located on the putative translocated region of chromosome 4AL were previously mapped on chromosome 7B in bread wheat (Somers et al. 2004; Semagn et al. 2006; Xue et al. 2008), which suggests that a segment of chromosome 7B moved to chromosome 4A.

The terminal portion of the long arm of chromosome 5A carries the locus *Xgwm6b*, which corresponds to the locus *Xgwm6a* on the long arm of chromosome 4B (Fig. 2b). The marker *Xdupw43* was positioned in the same region of chromosome 5A, while it was previously mapped on chromosome 4B in hexaploid wheat by Semagn et al. (2006).

DArT marker *wPt*-*8920* revealed two loci located on the long arm of chromosome 5B and on the short arm of chromosome 7B (Fig. 2c). In the same 7B region, there are two SSRs (*Xgwm537* and *Xwmc405*) that were previously mapped on chromosome 5B in the durum × ssp. *dicoccoides* map Omrabi/600545//Omrabi (*Xgwm537*) (Elouafi and Nachit 2004) and in bread and durum wheat maps (*Xwmc405*) (Somers et al. 2004; Mantovani et al. 2008).

A putative translocation that occurred between chromosomes 3B and 7B is also shown in the present study (Fig. 2d). Three loci that correspond to the EST-SSR marker *BJ213673* were mapped on the long arm of chromosome 7B and on the short arm of chromosomes 3A and 6B. Nevertheless, the same region of chromosome 7B carries three markers (*Xgwm112*, *Xwmc540,* and *Xcfd*6) that have already been shown to map on the corresponding region of chromosome 3B by Röder et al. (1998) and Somers et al. (2004), which suggests the occurrence of a T3B-7B translocation event.

Based on the same criteria adopted for previously described rearrangements, three more regions were identified that were characterized by a non-homoeologous relationship, even if two of them were based on only one marker. The segment of the short arm of chromosome 5B that contains the marker *Xwmc149* corresponds to the distal region on the long arm of chromosome 2B (Fig. 2e). The loci *Xgwm191* and *Xwmc363* were also mapped in this region; they were previously positioned on chromosome 5B in hexaploid wheat (Röder et al. 1998; Somers et al. 2004; Song et al. 2005), which suggests that this chromosome region was affected by a translocation.

The regions on chromosomes 1A and 6A reported in Fig. 2f, were collinear for the markers *wPt*-*9474,* *wPt*-*2632,* and *wPt*-*8773*. The possibility that a rearrangement involved these chromosome segments is further supported by the marker *Swes953*, which is located near the locus *wPt*-*9474b* on the long arm of chromosome 6A, and was previously mapped in wheat on chromosome 1B (Li et al. 2007).

Finally, two paralogous loci for the marker *Xgwm786* were mapped on chromosomes 5A and 6A (Fig. 2g). The short segment of chromosome 5A also includes *Xwmc150*, for which Somers et al. (2004) identified two loci on chromosomes 5A and 6A, in agreement with our results. A translocation T6B-5A can be hypothesized, as this region on chromosome 5A carries the locus *Xbarc180*, which was previously mapped on chromosome 6B in bread wheat (Song et al. 2005).

## Discussion

### Features of the durum wheat consensus map

The consensus map developed in the present study presents a much higher average density than that observed across the six individual maps. Values ranging from 11.8 cM/marker (Nachit et al. 2001) to 5.7 cM/marker (Mantovani et al. 2008) have been reported for published durum wheat individual linkage maps. Two consensus maps were previously developed for bread wheat by Somers et al. (2004) and Crossa et al. (2007). They mapped 1,235 and 1,644 markers, respectively, on the A, B, and D genomes. With respect to these maps, the durum consensus map herein described represents a large improvement. Due to the presence of regions which lack the statistical support for linking linkage groups belonging to the same chromosome, it was not possible to connect all the groups in a number corresponding to 14 chromosomes. The same feature characterized the map published by Trebbi et al. (2011). It is a durum wheat integrated map that they developed by merging two individual datasets. Even if the lengths of the two consensus maps were very similar, the consensus developed in the present study still has a greater number of markers (1,898 vs. 1,479), with better marker density (1.6 vs. 2.0 cM/marker). This is probably due to the integration of six individual maps that were derived from genotypes more genetically distant (11 durum wheat cultivars and one ssp. *dicoccoides*).

The high number of common markers, as well as the small differences in the recombination frequencies of the common markers across the different populations, can allow to position markers on a highly reliable reference map also in those regions that were poorly covered in the individual maps. Nevertheless, some regions with insufficient marker coverage (>20 cM) are still present in the consensus, which indicates a lack of polymorphism between specific parental pairs, due to recent co-ancestry, as suggested by pedigree data (data not shown). Alternatively, this can be due to the domestication bottle neck or a locally altered genetic versus physical distance ratio.

### Segregation distortion

In the present study, a similar proportion of skewed markers was found for DArT and SSR markers, according to Akbari et al. (2006), Peleg et al. (2008) and Mantovani et al. (2008). The major occurrence of skewed markers on the B genome as compared with the A genome is a common feature for durum wheat linkage maps (Peleg et al. 2008; Mantovani et al. 2008). An opposite behavior was seen for the bread wheat map reported by Semagn et al. (2006), in which the chromosomes of the A genome always had many more skewed markers than the B counterpart, except for group 6.

As markers with distorted segregation were clustered into specific regions, and as the same regions were identified in different backgrounds, this indicates that this phenomenon is linked to genetic factors and is unlikely to be due to genotyping or scoring errors. Distorted segregation can be explained by reduced fitness of gametes and zygotes that is determined by loci with lethal or sub-lethal effects linked to molecular markers (Foolad et al. 1995; Blanco et al. 1998). In our study based on RIL populations, the gametophytic selection probably has a limited role in segregation distortion, compared with other studies based on double haploid populations (Cadalen et al. 1997). Furthermore, chromosomal rearrangements can also explain segregation distortion (Faure et al. 1993). Indeed, the region on chromosome 7BS putatively involved in the translocation described in the present study contained skewed markers (Fig. 1). Nearly half of the regions that included skewed markers in this consensus map were located around centromeres, which are regions that generally show reduced recombination (Faris et al. 2000), according to most of the aforementioned studies. Knowing the positions of the skewed regions is very important in plant breeding, as they can affect the association marker-QTL and the obtaining of the desired recombinants.

### Analysis of colinearity between homoeologous chromosomes and structural rearrangements

Several of the 21 chromosomes of hexaploid wheat contain translocations of considerable sizes (Gale 1990), and the evolutionary evidence for translocations that have involved chromosome arms 4AL, 5AL, and 7BS has been firmly established (Chao et al. 1989; Naranjo 1990; Liu et al. 1992; Chen and Gustafson 1994, 1997; Blanco et al. 1998; Devos et al. 1995; Mickelson-Young et al. 1995; Nelson et al. 1995; Quarrie et al. 2005). This important cyclic translocation (4AL–5AL–7BS), evident also in our consensus map, has become an evolutionary signature of polyploidy wheat, which has conferred an adaptive advantage during the course of evolution (Devos et al. 1995). Possibly at the diploid level, chromosomes 4AL and 5AL exchanged terminal segments. Then, in tetraploid wheat, the distal portion of the chromosome 5A segment on chromosome 4AL was exchanged with a terminal segment from chromosome 7BS. Similar rearrangements have been documented in other grass genomes, such as rye, which has a translocation that corresponds to T4A–5A in wheat (King et al. 1994).

Furthermore, a pericentric inversion within chromosome 4A has also been reported in the literature (Miftahudin et al. 2004). Our results confirm this inversion (marker *Xwmc617*—Fig. 1) and suggest other inversions for the homoeologous groups 1 and 2 (markers *Xgwm403* on group 1 and *Xwmc51* on group 2—Fig. 1).

Moreover, the markers *Xwmc51* and *Xgwm71* show an additional locus on the same chromosome 2A, which suggests that a duplication of the region that comprises these markers has occurred during the evolution of the wheat genome. The presence of two distinct loci for the marker *Xgwm71* was confirmed by Röder et al. (1998) and Somers et al. (2004).

The other translocation, identified with the consensus map, on the long arm of chromosome 7B (Fig. 2d) could correspond to the translocation T3B:7B described for *T.* *dicoccoides* by Badaeva et al. (2007). As the rearrangements reported by these authors were cytogenetically identified, the translocation identified in the present study represents the description of a translocation T3B–7B with the molecular markers genetically mapped.

Three putative translocations are herein suggested that have never been described before, for chromosomes 2B–5A, 1B–6A, 5A–6B (Fig. 2). Out of seven translocation events proposed in the present study, five are inter-genomic, which suggests that recombination between homoeologous chromosomes might be common in polyploids following interspecific wheat hybridization (Wendel and Wessier 2000). Transposable elements might represent one of the mechanisms that form the basis of intergenomic rearrangements. Indeed, the union of two genomes into a single nucleus can be perceived as the introduction of ‘foreign DNA’ and can induce the activation of transposable elements during polyploidization, as has been shown in cotton and wheat (Wendel and Wessier 2000; Chantret et al. 2005). Interestingly, the markers *wPt*-*5964* and *wPt*-*7214* that are positioned within and near to a region on chromosome 7A that is involved in a translocation, correspond to sequences that code for retrotransposons (data not shown).

It is reasonable to expect that the ongoing wheat sequencing projects (http://www.wheatgenome.org) will reveal the evolution of the chromosome structure and the distributions and physical locations of additional breakpoints and rearrangements within all of the chromosomes of both hexaploid and tetraploid wheat, as revealed by a recent analysis of the gene content in chromosome 5A (Vitulo et al. 2011).

### The consensus map as a tool for advanced genetic studies in durum wheat

Consensus maps can be used to perform meta-QTL analysis (Haberle et al. 2009; Löffler et al. 2009), to combine genetic marker data and QTL characteristics (location, confidence interval, effects, and traits used for QTL detection) on a single map, as obtained from independent QTL mapping. This kind of study allows the optimal set of distinct consensus QTL (meta-QTL) to finally be estimated. Danan et al. (2011) clustered 144 QTL into 24 meta-QTL using a consensus map of potato that comprised 2,141 markers.

A limitation of QTL studies performed on individual biparental populations is often seen in the low number of molecular markers present in the region in which the QTL is identified. The projection onto high-density integrated maps of information derived from single population studies allows the QTL region to be enriched with many more molecular markers with respect to single populations. This thus represents an important advantage for fine QTL analysis, map-based gene cloning, transfer of QTL among different genetic backgrounds, and comparative studies between different genomes.

Linkage disequilibrium-based QTL analyses have been carried out for many agronomic traits related to grain yield and disease resistance in both common wheat (Crossa et al. 2007; Neumann et al. 2010) and durum wheat (Maccaferri et al. 2010). High-coverage integrated maps can have a positive effect on association mapping studies (Neumann et al. 2010; Trebbi et al. 2011). If unmapped markers have been used to genotype plants for association mapping, they can be tentatively ordered using the information provided by consensus maps. With more than 1,100 mapped DArT markers, the consensus map developed in the present study represents a valuable tool for association mapping of QTL on populations characterized with this high-throughput and cost-effective genotyping system.

The consensus map herein presented contains mapping data regarding 167 PCR-based molecular markers and 182 DArT markers for which the clone sequence is available (http://www.triticarte.com.au) and for which a match was found in the public databases using BLASTN and BLASTX searches (AM Mastrangelo, personal communication). Overall, a total of 349 markers related to expressed sequences were mapped in the present study, thus adding a functional value to the consensus map. The presence on a genetic map of markers derived from expressed sequences helps to associate candidate genes with identified QTL. Expressed-sequence-based molecular markers are also essential for colinearity studies among genomes, thus taking advantage of the available sequenced plant genome information, for faster fine mapping of QTL in species like wheat, for which the sequenced genome has not been released yet.

## Electronic supplementary material

Below is the link to the electronic supplementary material.

Online resource 1 (XLSX 12 kb)

Online resource 2 (XLS 165 kb)

Online resource 3 (PDF 2031 kb)

Online resource 4 (XLS 35 kb)
