# Supplementary material for: A high-density consensus map of A and B wheat genomes
Source: Theor Appl Genet. 2012 Aug 8;125(8):1619–38. doi: 10.1007/s00122-012-1939-y (PMC3493672; doi:10.1007/s00122-012-1939-y)
Supplement: Supplementary file 3 — Online resource 3 (PDF 2031 kb) [file 122_2012_1939_MOESM3_ESM.pdf]

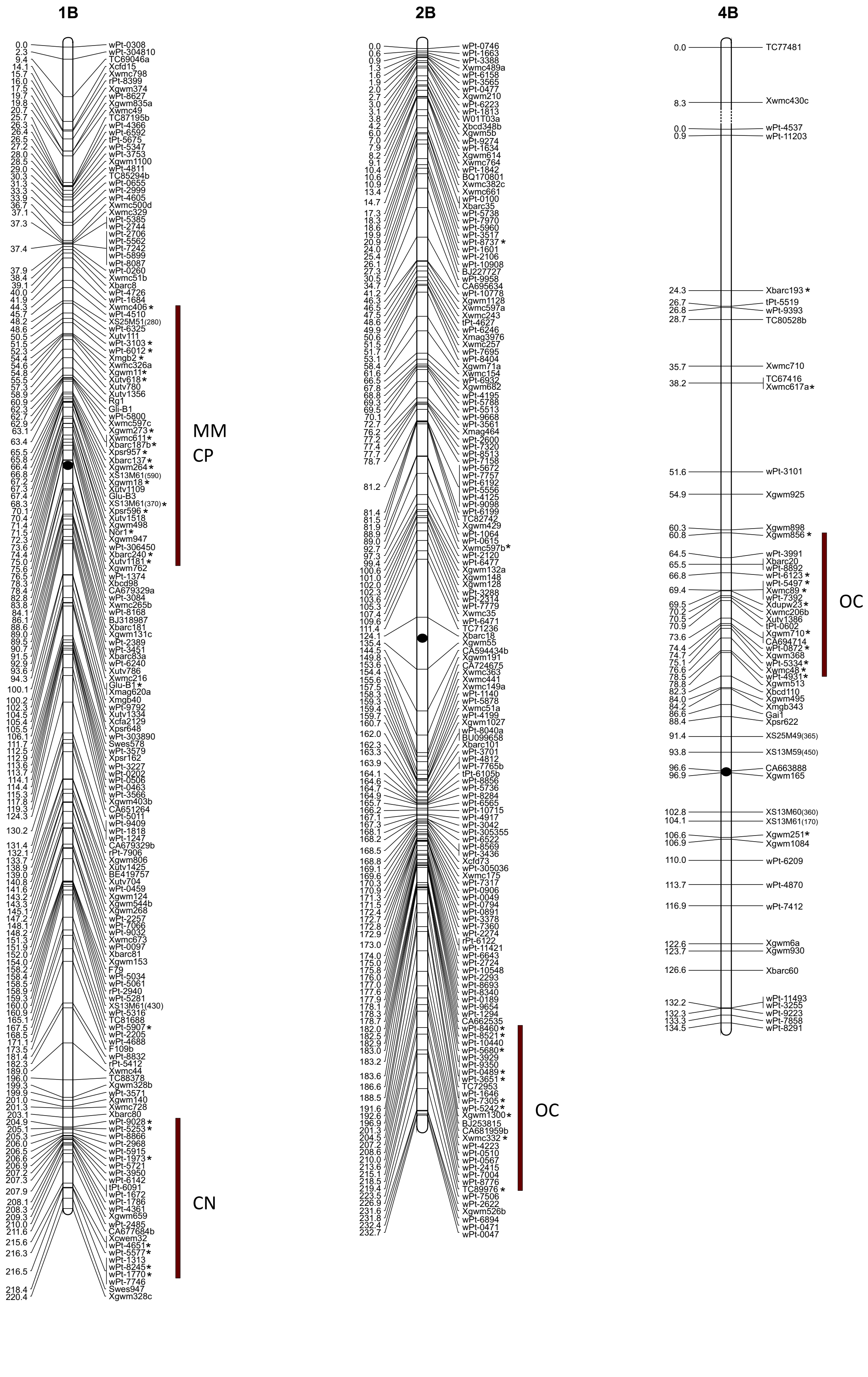


# A HIGH-DENSITY CONSENSUS MAP OF A AND B WHEAT GENOMES

Theor. Appl. Genet.

Daniela Marone, Giovanni Laidò, Agata Gadaleta, Pasqualina Colasuonno, Donatella B.M. Ficco, Angelica Giancaspro, Stefania Giove, Giosue' Panio, Maria A. Russo, Pasquale De Vita, Luigi Cattivelli, Roberto Papa, Antonio Blanco, Anna M. Mastrangelo
Corresponding author: Anna Maria Mastrangelo, CRA-Cereal Research Centre SS 16 - km 675 71122 Foggia, Italy.
E-mail: annamaria.mastrangelo@entecra.it

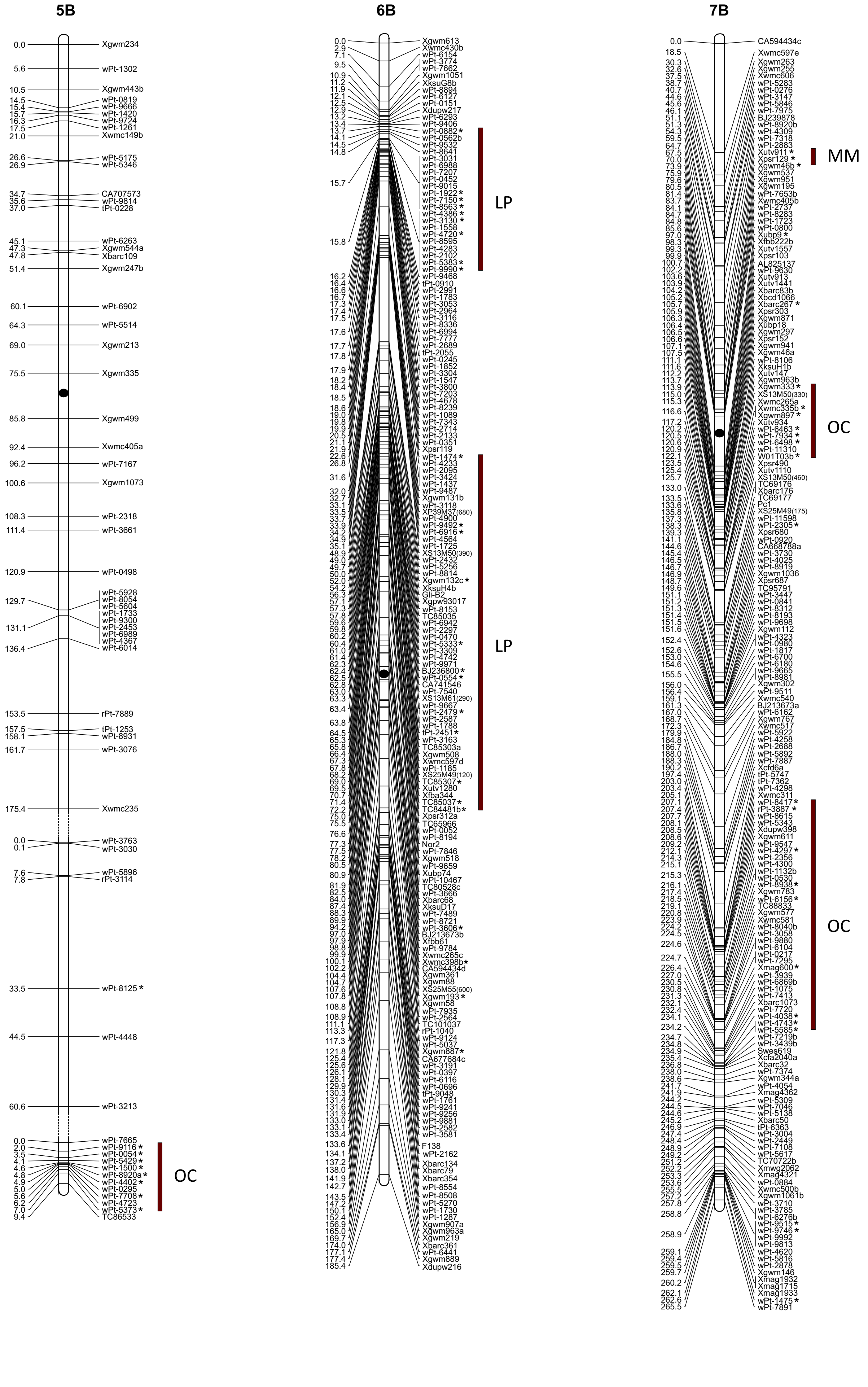
5B
6B
7B
OC
LP
LP
MM
OC
OC
